# Applications of Artificial Intelligence for Heat Stress Management in Ruminant Livestock

**DOI:** 10.3390/s24185890

**Published:** 2024-09-11

**Authors:** Ebenezer Binuni Rebez, Veerasamy Sejian, Mullakkalparambil Velayudhan Silpa, Gajendirane Kalaignazhal, Duraisamy Thirunavukkarasu, Chinnasamy Devaraj, Kumar Tej Nikhil, Jacob Ninan, Artabandhu Sahoo, Nicola Lacetera, Frank Rowland Dunshea

**Affiliations:** 1 Rajiv Gandhi Institute of Veterinary Education and Research, Kurumbapet, Puducherry 605009, India; binunirebez.e@gmail.com (E.B.R.); mv.silpa@gmail.com (M.V.S.); drnikhilkumartej@gmail.com (K.T.N.); ninanjacob@river.edu.in (J.N.); 2 ICAR-National Institute of Animal Nutrition and Physiology, Adugodi, Bangalore 560030, India; drcdeva@gmail.com (C.D.); sahooarta1@gmail.com (A.S.); 3 Department of Animal Breeding and Genetics, College of Veterinary Science and Animal Husbandry, Odisha University of Agriculture and Technology, Bhubaneshwar 751003, India; gnazhal99@gmail.com; 4 Department of Veterinary and Animal Husbandry Extension Education, Veterinary College and Research Institute, Tamil Nadu Veterinary and Animal Sciences University, Namakkal 637002, India; dthirunavukkarasu@gmail.com; 5 Department of Agriculture and Forest Sciences, University of Tuscia, 01100 Viterbo, Italy; nicgio@unitus.it; 6 School of Agriculture, Food and Ecosystem Sciences, Faculty of Science, The University of Melbourne, Parkville, Melbourne, VIC 3010, Australia; fdunshea@unimelb.edu.au

**Keywords:** artificial intelligence, deep learning, heat stress, machine learning, neural networks, ruminant livestock

## Abstract

Heat stress impacts ruminant livestock production on varied levels in this alarming climate breakdown scenario. The drastic effects of the global climate change-associated heat stress in ruminant livestock demands constructive evaluation of animal performance bordering on effective monitoring systems. In this climate-smart digital age, adoption of advanced and developing Artificial Intelligence (AI) technologies is gaining traction for efficient heat stress management. AI has widely penetrated the climate sensitive ruminant livestock sector due to its promising and plausible scope in assessing production risks and the climate resilience of ruminant livestock. Significant improvement has been achieved alongside the adoption of novel AI algorithms to evaluate the performance of ruminant livestock. These AI-powered tools have the robustness and competence to expand the evaluation of animal performance and help in minimising the production losses associated with heat stress in ruminant livestock. Advanced heat stress management through automated monitoring of heat stress in ruminant livestock based on behaviour, physiology and animal health responses have been widely accepted due to the evolution of technologies like machine learning (ML), neural networks and deep learning (DL). The AI-enabled tools involving automated data collection, pre-processing, data wrangling, development of appropriate algorithms, and deployment of models assist the livestock producers in decision-making based on real-time monitoring and act as early-stage warning systems to forecast disease dynamics based on prediction models. Due to the convincing performance, precision, and accuracy of AI models, the climate-smart livestock production imbibes AI technologies for scaled use in the successful reducing of heat stress in ruminant livestock, thereby ensuring sustainable livestock production and safeguarding the global economy.

## 1. Introduction

**Figure 1:**
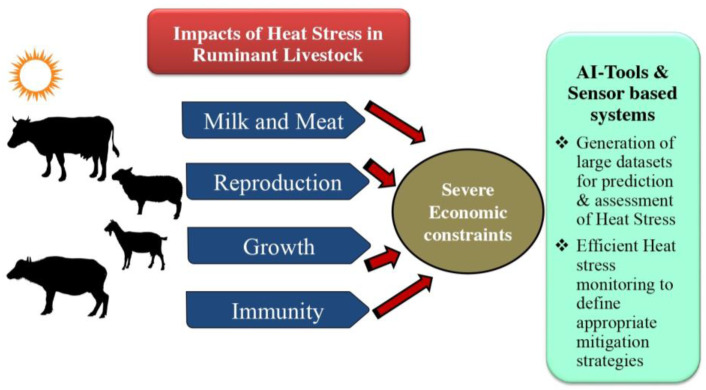
Significance of AI tools in monitoring the productive performance of ruminant livestock and reducing the heat stress-associated economic loss.

Climate change is one of the major threats to the survival and sustainability of climate sensitive sectors across the globe. The Intergovernmental Panel on Climate Change (IPCC) has reported that global warming will continue to increase in the near future (2021–2040) and is more likely to reach 1.5 °C, even under the very low greenhouse gas (GHG) emission scenario, and likely or very likely to exceed 1.5 °C under higher emissions scenarios [1]. As global warming progresses and the magnitude of temperature extremes increase, it has been forecasted that heat stress affecting livestock will be a major concern affecting the economy. The increased demand for livestock products in the changing climatic condition has become a pressing challenge. The climate change-induced exceeding temperature thresholds leading to decreased productivity in ruminants results in severe economic loss to the farmers [2]. This necessitated a radical change in the production systems by adoption of sustainable steps on both the production and consumption fronts to decrease food insecurity. In addition, ruminant livestock contributes to sustainability by utilising uncultivable land for food production, converting non-human energy and protein sources into highly nutritious animal-sourced food, and provides a living for millions of people worldwide [3]. Thus, directly and indirectly, ruminant livestock rearing contributes to the global economy. Therefore, sustainable livestock farming is a vast challenge demanding advanced, cutting-edge climate-resilient management practises. Figure 1 describes the economic losses incurred in livestock farms as a result of heat stress and signifies the importance of AI tools in reversing this loss. 

**Figure 2:**
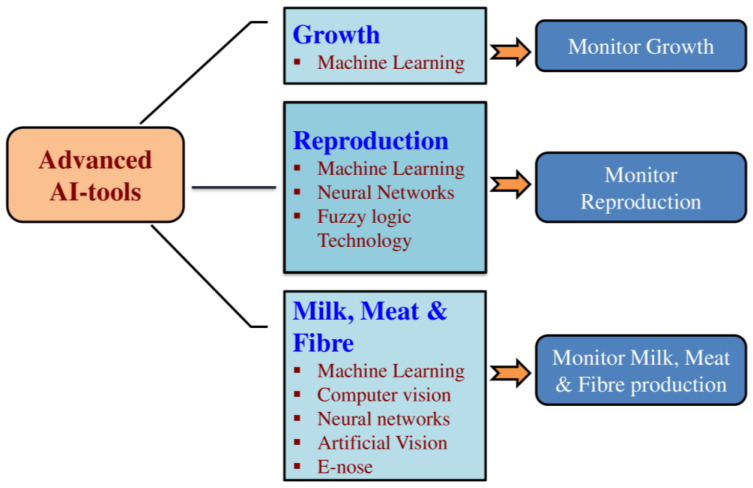
AI tools used for monitoring productive performance in livestock.

The era of climate-smart livestock farming has stepped into a new phase as we enter the digital age. Presently, numerous studies on sensors, data collection and processing, modelling tools and algorithms, artificial neural networks, deep learning (DL), machine learning (ML), etc. have been conducted in an effort to address issues with animal identification, behaviour detection, disease monitoring, environmental control, and other related problems in livestock production systems. Artificial neural networks (ANNs), Convolutional neural networks (CNNs), DL, adaptive neural fuzzy inference systems (ANFIS), ML, and pattern recognition (PR) are some of the Artificial Intelligence (AI) models that are commonly used for modelling, prediction, and management of animal farming [4]. It has been established that, in this climate challenging situation, identification of sustainable animals using phenotypic markers identified and integrated using several ML approaches would create a promising and sustainable livestock sector [5]. The AI technologies can help to continuously monitor the animals and to implement cost effective strategies to improve their welfare, as well as accurately predict disease occurrences resulting in reduced use of drugs and agro-chemicals contributing to sustainable livestock farming [6,7]. Further, the potential application of these energy-efficient technologies to decrease resource exhaustion and improve the efficiency of animal management, which in turn contribute to economic stability with very minimal environmental impacts, results in sustainable livestock production [8]. Figure 2 describes the various AI tools used for monitoring productive functions in livestock. Thus, a wide range of user-friendly heat stress managing AI technologies are developed to communicate with livestock farmers and disseminate information about appropriate animal health practises, feeding practises, and management techniques. Further, the AI technologies improve profitability by providing real-time information about the animal health status, aiding in early disease diagnosis, the tailoring of accurate feeding plans, and resource scheduling, thereby contributing to cost-effectiveness [9]. These advanced interventions improving the profitability of livestock farming would thus empower the rural livelihood. This review is therefore an attempt to collate information pertaining to the various AI applications, which aids in heat stress management for sustainable ruminant livestock production. Table 1 describes the economic values of different ruminant livestock.

## 2. Significance of AI in Modern Ruminant Livestock Production

By 2050, it is predicted that the demand for animal products will increase by between 60% and 70% [10]. Further, the breakdown of climate has limited resources and hence effective ruminant livestock production strategies have been postulated to be crucial. In this regard, constructive evaluation of production, welfare, and the health of ruminant livestock farms have been employed by adoption of advanced and developing digital technologies. This has overcome the limitations caused by manual labour involvement in rearing ruminant livestock. This also implies that manual intensive animal husbandry practises may no longer be adequate in livestock production systems in this climate change scenario. Thus, devising effective strategies and methods to assist in gaining larger returns in animal production is vital. AI in particular is widely employed and popular due to its ability to efficiently and continuously monitor livestock and their environments. It helps farmers and producers to make more informed decisions about their operations and better comprehend animal behaviour and discomfort to effectively manage and improve production [11]. In addition, these tools can be adopted with ease, as computing power is now readily accessible to a wide range of livestock producers.

Accurate behavioural monitoring using sensors has the potential to provide a system for the early diagnosis and assessment of heat stress in the livestock production sector. It is possible to create a completely automated system for non-invasive data collection, processing, and interpretation using promising methods of AI application with remote sensing and ML modelling techniques. In order to forecast heat-stressed animals, methods that use light detection and ranging (LiDAR) with visual, thermal, multispectral, and hyperspectral camera inputs can be adopted to predict relatable factors [12]. This can help by offering valuable details on the intensity of the factors linked to heat stress in livestock. 

An advanced approach of using ML algorithms to assess the effect of environmental stressors on the physiological responses of the animal has been reported, wherein ML models are employed to rate the impact of environmental factors on dairy cows’ physiological reactions [13]. Further, the livestock sector is undergoing a robust advancement and major digital transition that has been prioritised and prompted by the recent technological bloom. Many operations in smart livestock farming use AI to quantify animal stress objectively and to evaluate its effect on animal production and welfare. Thus, the potential applications of AI in monitoring, evaluating and quantifying stress factors by overcoming the challenges related to the complex nature of animal production systems adds significance in this era of modern livestock production. Further, it adds value in assisting farmers to raise earnings by enhancing animal production. 

## 3. Evaluation of Production Parameters of Heat-Stressed Animals Using AI Technologies

Unfavourable climatic variations are anticipated to be one of the primary barriers to ruminant livestock production in the tropics. In particular, high ambient temperatures are poised to be the main factor limiting animal productivity. Heat stress, being a significant challenge in the livestock production units, alters the biological systems of the animals, affecting their growth, reproductive capacity, as well as the quantity and quality of milk, meat, and fibre. In order to construct dynamic, self-calibrating model-based systems that might enable real-time evaluation and minimization of heat stress, robust monitoring systems are required. Moreover, technologies with novel AI algorithms are the current mandate of the hour. These AI technologies might improve the scope of the evaluation of production variables by commercial producers and livestock owners and thereby minimise the incurring production losses. Due to its wide application, the development of AI technologies is being investigated for its potential to improve farm animals’ productive performance. 

### 3.1. Growth

Broadly, environment, nutrient availability and genetic factors are the intricate variables that might affect the growth of an animal. Heat stress has been shown to have a negative impact on animal growth performance in tropical and sub-tropical regions around the world. Heat stress has a noticeable effect on body weight, average daily gain, growth rate, feed consumption, production efficiency or weight gain per unit of feed energy [14]. A decrease in the body condition score (BCS) of animals under stress could be attributed to body reserve depletion [2]. Many studies have reported the use of AI in the measurement of body weight and BCS. For instance, a research finding estimated the body volume of mature beef cows from depth images, quantified body weight and metabolic weight from image-projected body volume, and classified the BCS from image-obtained measurements using an ML-based approach [15]. By adoption of sophisticated ML algorithms like ensemble models and transfer learning, researchers were able to predict the BCS of dairy cows with high accuracy [16]. In order to determine the relationships between climatic factors and growth parameters like the BCS, improvements have been made to the current prediction models with advanced and automated technologies. It has been reported that PR models can be utilised to process and analyse information from pictures, videos, and sounds in a manner similar to how the brain functions through biological perception and computer realisation in order to characterise growth performances [4]. These models can help identify animals that exhibit a significant change in growth performance due to heat stress. The prediction of body weight and BCS using AI technologies would be a novel, low-cost approach that facilitates livestock producers with limited or no access to weighing facilities to proactively monitor and manage the livestock herds. In addition, BCS determination acts as a significant management tool for determining the energy reserves of the animal under changing and challenging environmental conditions. Thus, the AI based BCS assessment can be effectively used to monitor the desired output of a livestock farm, providing a cost effective approach to monitoring the welfare of the animals [17,18].

Specifically, a high temperature environment causes a change in feed resource availability, which is another stressor that impacts growth variables. In this feed inadequacy state, an evaluation of the feed conversion efficiency (FCR) of animals using AI technologies would serve as a significant determinant of growth performance. It has been reported that a novel system that ranks cows according to their feed conversion efficiency in commercial farms has proven beneficial in this regard [19]. The three main components of the system are as follows: the weighing system, which uses a single load cell suspended to measure feed mass; the image-based cow identification system replacing Radio Frequency Identification with cameras erected over the feeding area; and an image processing algorithm that uses the collar numbers of the cows to identify them [19]. This method can be accurate in identifying efficient, thermo-tolerant animals. Thus, by understanding the alterations in feed efficiency, it is possible to differentiate possible variations in the thermo-tolerance potential of animals under heat stress. Generally, it is expected that heat stress reduces the feed conversion efficiency in animals, and the thermo-tolerant breeds are considered to have a higher FCR. This would assist producers in replacing inefficient animals and in breeding more heat-tolerant, efficient animals that can be profitable for livestock producers. Feed efficiency, a multifactorial functional trait that reflects the energy balance of an animal, and which determines its overall productivity, can thus be evaluated.

The ML approaches can thus aid in measuring the altered circulating metabolites in a heat-stressed animal. For instance, a study in buffalo heifers employed ML algorithms for predicting feed conversion efficiency, using predictor variables like haematological parameters and average daily gain, wherein Partial Least Square Regression (PLSR) models were created using least square means. The study concluded that higher feed conversion efficiency measures were found to be influenced by Insulin like Growth Factor-1(IGF-1) and its interactions with other blood parameters [20]. Further, the magnitude of the estimated interaction effects of blood parameters in relation to feed conversion efficiency may aid comprehension of the intricate dynamics of blood parameters for growth under heat-stressed conditions. 

### 3.2. Reproduction

Extreme climatic conditions will place multiple stressors on livestock animals, reducing their reproductive capacity. In this regard, heat stress contributes a major part in increasing the susceptibility of reproductive systems of the ruminant livestock to heat waves. The heat-stressed ruminant, in an attempt to avoid hyperthermia, reveal adaptive changes in physiological functions. Subsequently, these changes, and/or hyperthermia, cause modifications of non-adaptive physiological functions like reproduction by negatively influencing the hypothalamo–pituitary–gonadal axis [21]. Furthermore, it has been documented that heat stress alters follicular dynamics and oestrus expression, reduces conception rate, increases embryo mortality, impairs luteal function, disrupts gonadotrophin and oestradiol secretions, and increases the frequency of silent oestrus and the development of ovarian cysts in dairy cows [21]. Thus, the quantification of changes in physiological variables is critical in assessing heat-stressed animals. Hence, responsible AI applications have aided in the development of advanced and emerging technologies for livestock assessment by extracting key physiological parameters associated with reproductive function. 

It is well established that endocrine dynamics become altered in heat-stressed animals, resulting in adverse alterations to the oestrus cycle [22]. In this regard, adoption of advanced AI technologies will aid in assessment of heat stress-associated alterations in oestrus dynamics in ruminant livestock. For example, an efficient heat detection technique developed in cattle using supervised ML, based on continuous monitoring of vaginal temperature (VT) and vaginal conductivity (VC), offers greater advantages due to its flexibility in integrating numerous characteristics [23]. In addition, this AI-based wearable wireless vaginal sensor device acts as an accurate and efficient oestrus identification model with high accuracy and sensitivity, identifying oestrus in real-time [23]. Further, evidence portrays the usage of fuzzy logic technology adopted for classification of oestrus impulses using a model-based detection approach employing the circular structure of oestrus [24]. 

To predict oestrus in breeding cows, another study compared the performance of three ML algorithms, i.e., expectation maximisation, random forests, and CNNs, based on three behavioural patterns of breeding cows, i.e., oestrus start, peak oestrus activities, and oestrus finish. The system was designed and put into place to analyse the 3-axis acceleration data from Internet of Things (IoT) sensors, and, specifically, the CNN performed exceptionally well in the trial when compared to the traditional machine-learning techniques [25]. Evidence has indicated than an efficient AI-based technique for the assessment of the oestrus cycle is the use of an ML algorithm based on the correlation of milk parameters, including density, pH, SNF, specific gravity, fat content, and the age, quantity, and breed of the cow. Decision tree classifiers and other ML algorithms receive the chosen parameters as input. Due to higher accuracy and performance, it acts as an efficient classifier and a straightforward technique for identifying oestrus using milk characteristics in animals [26]. The animals under heat stress conditions also experience silent or indistinct oestrus. However, the challenging part is the identification of silent oestrus in animals, as there are no outward manifestations of mounting behaviours [27,28,29]. As a result, these AI-based solutions may be used to evaluate heat stress effects on the oestrus cycle effectively.

It has been established that thermal injury to oocytes causes morphological abnormalities, oxidative stress, nuclear fragmentation, and mitochondrial dysfunction, as the ovarian pool of oocytes is most vulnerable to heat [30]. Considering these heat stress-associated disruptions, it is crucial to assess the oocyte quality for indirect evaluation of heat stress-induced damages. It has been suggested that a qualitative identification of oocytes can be performed by applying advanced methods of neural analysis of graphic data. Thus, use of ANNs for classifying data in graphic form [31] can be employed, and the adoption of neural image analysis might enable effective identification of the quality of oocytes [32] to assess heat stress in animals. Further, as the number of oocytes to be inspected is huge, involving a laborious and complex process, supervised ML methods like random forest have been reported to make the analysis task standardised, reducing the inter-subject variability. It is also a semi-automatic framework to predict and grade the class of oocyte based on feature-based classification utilising random forests after multi-object parametric segmentation on the obtained microscopic image [33].

Based on AI techniques such as genetic algorithms (GAs) and ANNs, a study has revealed its potential use in digital-image processing to assess bovine blastocyst characteristics. The assessment is based on objectively classifying embryo quality by using mathematical variables that have been extracted from the obtained digital image. Following automated feature extraction of the images, the output is fed into a supervised learning process [34]. These advanced AI-based tools might act as a robust method for embryo analysis under heat-stressed conditions. 

It is well established that the conception rate (CR) declines during the summer season [33,35]. Three distinct back propagation algorithms utilising the temperature humidity index (THI) and infrared thermography (IRT) temperature as inputs and manually recorded (rectal temperature) RT as targets were used to develop ANN models. The created approach would thus have the potential to act as a rapid and economical method for detecting heat stress with minimum constraint and tracking body temperature in real-time [36]. Moreover, a study reported the use of a type of ML algorithm, the alternating decision tree, to identify factors that affect the first-service conception rate in Holstein cows using a comprehensive dataset that includes details about management, housing, labour, nutrition, genetics, and climate for individual farms and cows on these farms [37]. This evidence suggests that AI-based tools can be utilised effectively in predicting the effect of multiple management practises and environmental factors on the reproductive performance.

Heat stress also has a significant influence on embryonic growth and survival, and it is validated by a study wherein mild hyperthermia had a profound effect on embryos [30]. Accordingly, heat stress is a significant risk factor for the development of intrauterine growth restriction (IUGR). Therefore, heat stress effects on embryos can be assessed indirectly by identifying the potential molecular markers of IUGR. In this regard, a study reports the use of a combination of ML algorithms to find the molecular markers of IUGR in sheep or IUGR-related hub genes (IUGR-HGs), which is then integrated into the study model of ANN for the early detection and evaluation of IUGR [38]. In future, as in human medicine, the use of ML approaches can be targeted, as multi-parametric ML approaches show notable improvements over univariate analysis in terms of specificity and sensitivity. In addition, it is suggested that the adoption of AI and ML techniques can act as a screening method to identify IUGR foetuses [39]. 

Moreover, it is vital to evaluate the reproductive performance of an animal under heat stress conditions using AI-enabled techniques. In this regard, a study finding validates the use of Matlab Fuzzy Toolbox to design a system which provides information about the breeding performances of cows, and thereby the reproductive efficiency can be evaluated [24]. These advancements can thus be readily adopted in assessment of heat stress effects on the reproductive systems of ruminant livestock. 

### 3.3. Milk, Meat, and Fibre

It is widely established that heat stress has an impact on a number of facets of animal productivity, as measured by milk [40], meat [41], fibre yield [42] and quality, inflicting heavy economic losses for the resource-poor farmers. These negative impacts of heat stress necessitate an advanced and robust assessment tool for enhanced production. In this regard, AI-enabled tools based on auto-learn models are suggested to play an integral role in evaluation of heat stress impacts on production variables. 

A study finding reports that a significant AI system utilises detailed information from a robotic dairy farm to enhance or sustain a desired degree of milk quality by mitigating heat stress [43]. In the same study, it has been suggested that the developed ML models can provide real-time information on milk volume and quality and real-time data on a per cow basis for effective heat stress management. Moreover, the study concludes that only minor technical improvements will be needed to use AI in dairy farms using the ML models described in the study [43]. Additional evidence regarding the application of AI in dairy farms reports the use of four ML algorithms (penalised linear regression, random forests, gradient-boosted machines, and neural networks) to assess the influence of environmental heat stressors on the physiological responses of dairy cows. These algorithms remove subjectivity and aid in ranking environmental heat stressors, which readily supports producers in implementing evidence-based remedies before expected stressful environmental circumstances arise, thereby improving production [13]. 

An AI-powered tool has been studied in a dairy farm wherein the algorithms are designed to extract information from videos and infrared thermal imagery (IRTI) [43]. The model can be practically applied using red, blue, and green (RGB) camera systems to obtain all the proposed targets, including eye temperature, which can also be used to model animal welfare and biotic/abiotic stress. The study is based on implementing computer vision algorithms to build an ML model based on regression fitting to predict eye temperature, daily milk productivity, cow milk productivity, milk fat, and milk protein with no signs of over fitting [44]. These models can be applied through manipulation of the stressful environment in conventional and robotic dairy farms [43].

There are multiple advantages to ML algorithms that are commonly suggested by statisticians due to their providing accurate and superior results; these algorithms learn from the data supplied and additionally reduce the chance of bias by the researcher’s hypothesis, as in traditional analytical methods [13]. Moreover, it is well established that ML is a potential method in dairy research that can be utilised in full measure to enhance decision-making. Since cows are multifactorial systems, ML algorithms may evaluate integrated data sources to characterise and, in the end, enable cow management based on all pertinent influencing elements [45]. For instance, a study on lactating Holstein cows reported the use of data on the age, body mass (BM), days in milk (DIM), daily milk yield (DMY), and milk temperature (MT) of animals from a robotic dairy farm for a period of five years along with ambient temperature data from the local weather station to determine dynamic heat stress thresholds with different stages (comfort stage, milk heat stress, effective heat stress, and critical heat stress). The decision tree ML model was used to identify and categorise these thresholds for individual animals. The categorization reached an excellent accuracy of 79–94%, which can be used as an alert for heat stress to implement cooling methods during the lactation phase [46]. A study trained and tested a model based on ML techniques using a random forest algorithm in assessing the milk yield of individual animals in relation to the varying environmental conditions. The study suggested its use in milk yield predictions by the model, as the average relative prediction error is minimal [47]. 

AI-powered tools can be used as prediction models and in studying relationships between variables. In this regard, a relationship model of physiological, environmental, and milk productivity by adopting AI in a study has proven beneficial. The model involves data collection and storage in a database, following which training and validation using a Back Propagation Neural Network (BPNN) with genetic algorithm optimisation is enabled. Consequently, this system has created an intelligent tool that could accurately forecast milk production in any kind of physical or environmental state. In the same study, based on sensitivity analysis, the factors that affected milk production were body temperature, environment temperature, relative humidity, and heart rate due to heat stress, as presented by the BPNN model. Moreover, the same study validated that effective use of AI models in determining the relationship between physiological, environmental, and milk productivity will be practically applicable in deciding the best management practise for improved milk production [48]. 

A research finding validated the use of empirical data on medium and maximum air temperatures around the milk sheds of cows to develop a neural prediction model in evaluating the milk yield under varied thermal conditions and suggested the model to be a practical guide for forecasting short-term milk yield in cows [49]. Thus, neural networks can act as an adaptive system to predict and evaluate the impact of heat stress in addition to aiding as support to chart out effective management measures. Another method to assess heat stress is based on the physical activity of cows. In this regard, a study involving the use of cluster analysis revealed that the temperature and humidity had an impact on the physical activity of cows milked in automated milking systems. The results revealed that the physical activity was lower in winter season, whereas lower humidity levels increased the animal’s physical activity [50]. These clustering techniques relying on unsupervised ML can thus be adopted in heat stress assessment. 

The negative impact of heat stress on meat production is known to be due to pathogen colonisation due to the high ambient temperature and humidity acting as favourable conditions in meat and its by-products [51]. Thus, assessment of meat quality is crucial to improving management practises and safety aspects during extreme environmental conditions. A study finding reveals a robust, rapid and non-destructive method for beef quality evaluation based on an artificial vision technology. The study utilised a fuzzy ARTMAP classifier model that classifies meat samples based on total microbial population into unspoiled and spoiled [52]. 

Another research finding reveals a tool based on processing digital images. In the study, deep CNN architecture has been modelled and trained to categorise images as “fresh” or “spoiled” following a pre-processing procedure on the recorded images [53]. In recent years, researchers have studied the application of AI technologies in meat quality assessments in regard to elements such as evaluating of the sensory qualities of meat (such as freshness, tenderness, colour, and the texture of meat) [54]. Moreover, the prediction of physical and chemical markers of meat quality, like pH, shear force, water retention, protein, and moisture content, have been evaluated using AI tools [55]. Another advanced tool for assessing meat spoilage is by the use of electronic olfaction. An electronic nose, which comprises an array of chemical sensors with partial specificity and an appropriate pattern recognition system, is capable of recognising simple or complex odours [56]. Therefore, these exciting tools can be used to assess heat stress-induced alterations in meat quality. There is a lot of established evidence elaborating on the impact of heat stress on meat quality and quantity. However, carcass evaluations were conducted using conventional methods until the introduction of AI in the meat industry. The few AI tools that have been described in this review portray the potential use of these tools in assessing heat stress-associated changes in meat quality and quantity. But there is no established evidence of AI tools assessing the meat quality of heat-stressed animals. However, this suggestion can be considered a critical research gap that can be addressed soon. 

It is well established that heat stress conditions present a significant risk, hindering the efficiency of wool production and thereby inducing severe economic losses for the wool industry [57]. Hence, forecasting the wool production using cutting edge AI applications would provide better returns for wool producers and allow them to remain competitive in the fibre market in this climate change scenario. It is evident that a variety of environmental conditions and management techniques, in addition to genetics, have a direct or indirect impact on the amount and quality of wool produced, and these impacts must be taken into consideration in forecasts [58]. Fibre diameter and clean fleece weight are affected by climate and management and are some of the main indicators of wool returns. Thus, a study reported the most efficient algorithm to predict adult greasy fleece weight, adult clean fleece weight, adult fibre diameter, adult staple length, and adult staple strength using flock-specific environmental data and yearling lamb phenotypic data [58]. Thus, similar models using best performing algorithms in assessing the effect of climate can be developed in future, which will help wool producers to plan and set up an efficient wool production system in this climate-breakdown scenario. 

## 4. Evaluation of Genetic Potential of Heat-Stressed Animals Using AI Technologies

Presently, in the face of climate change, heat stress is considered to be an important challenge especially in regard to tropical livestock production systems. Heat-stress alleviation strategies are multi-faceted, involving efficient management, nutrition, and genetic approaches [59,60]. In regard to the genetic measures, accurate methods to evaluate the genetic performance of animals with thermo-tolerant traits is a priority. Vast volumes of data need to be saved every day due to the creation of “big data” sets as a result of improvements in genetic technologies [61]. In this regard, AI systems that are frequently used to evaluate large volumes of data to generate predictions can be adopted, and this might have important ramifications in planning mitigation strategies. In addition, these large datasets may reveal a defined modification in the genome of the animal, which enables it to adapt to a variety of environmental conditions and thus gives clues about the adaptation of a species. However, the information on adaptation is a combination of heterogeneous and homogeneous data types, and it may be difficult to determine how one attribute relates to another. Thus, to go above the limitations of conventional linear models, it has been reported that AI and ML techniques are being utilised more and more to extract information from this kind of data [62].

The genetic improvement for thermo-tolerance is not frequently used in the dairy sector despite the fact that the currently employed genetic measures are sound. However, it is recognised that the selection of heat tolerance traits is feasible since it is well acknowledged that genetic diversity is linked to the performance of animals under heat stress [63]. Genomic selection is a novel approach to evaluate genetic performance and is being applied extensively in various regions due to its various advantages over conventional methods. Accelerating the genomic selection process using AI-enabled technologies would thus be beneficial. ML has gained traction in livestock genomic prediction, just as its popularity has surged in other domains. The capacity of ML algorithms to identify patterns in massive, unstructured datasets, even when information about some possible explanatory factors is missing, is well recognised. Moreover, ML algorithms are flexible and beneficial when combining vast amounts of genomic and phenotypic data with biological information from planned trials to forecast the breeding values of selection candidates [64]. 

The results of an investigation reveal the extreme flexibility of ML methods using a random forest algorithm by identifying possibly additive and epistatic quantitative trait loci (QTL) influencing residual feed consumption in dairy calves [65]. This property can thus be exploited in heat stress scenarios to identify the QTL influencing thermo-tolerance in ruminant livestock. It has been established in a study finding that ML has enormous promise for improving genetic selection and dairy herd management, especially when it comes to DL techniques for multi-layer ANN implementation [64]. ANN has been proposed as a potentially useful device for marker-based genomic predictions of complex traits in animal breeding. Further, the study revealed significant ANN use in regard to noisy and high-dimensional data, mainly when the trait’s genetic architecture is unclear, thus making ANN a potent tool for making non-linear genome-enabled predictions [66]. Moreover, a genome-wide panel of dense markers is used in genomic selection to increase the likelihood that every QTL is in linkage disequilibrium with at least one SNP. The genomic estimated breeding values (GEBVs) are then evaluated [67]. Thus, genomic selection remains an attractive option for selecting animals based on their heat tolerance, GEBVs, and other traits [68]. In this regard, AI-directed tools like ANNs and neuro turbid systems have been reported to help estimate breeding values in dairy cattle [69]. 

Evaluation of heat-tolerance and selection based on a thermo-tolerance trait is considered a significant measure. In this regard, study findings have shown the development of genomic breeding value for heat tolerance in Australian dairy cattle with components such as a decline in milk, fat, and protein per unit increase in THI when THI increases above the threshold of 60 [63]. In addition to studying the effects of heat stress on the economically important milk production trait, other traits like somatic cell score (SCS), which is related to mastitis, and the milk fat to protein ratio (FPR) trait, which is an indicator for evaluating negative energy balance and ketosis, are intriguing to study alongside heat stress in order to enhance the genetics of production parameters [70]. The application of AI- in these processes is vital, and robust methods integrated with AI-powered tools can be adopted for evaluating the performance of animals under heat-stressed conditions to strategize selection programmes and sustainably resolve heat-stress-associated production losses. However, selecting markers accordingly is crucial, and it is important to highlight the significance of algorithm selection for efficient genomic prediction [71]. 

Over many generations, certain native breeds raised in harsh environments, such as high temperatures and humidity or droughts, have adapted, making them an excellent resource for breeding. In this alarming climate change scenario, selecting animals resistant to temperature extremes is crucial. It is critical to comprehend the biological processes associated with the adaptability of indigenous breeds, particularly identifying the genomic areas and genes that govern such processes. In this facet, the selection of climate-resilient animals can be accelerated by genome-wide association studies (GWASs) that aid in identifying genes governing climate adaption features (e.g., effective thermoregulation, feed utilisation, and immune system) [72]. ML algorithms have become widely used because they effectively create prediction models in situations with more features than samples. In addition, GWAS statistical testing is the conventional method used to find the variants that affect the desired phenotype; however, due to their diverse range of applications, ML methods have great potential for improving our understanding of the impacts of these variations [73]. 

The ML algorithms prioritise enhancing the precision of predictions at the individual subject level [74]. Recently, ML techniques have started to complement or even replace standard statistical genetic approaches [75]. Algorithms, including ensemble, neural networks, regression, and classification, have been used for GWASs with many applications. Further, significant single-nucleotide polymorphisms (SNPs), disease risk assessment and prediction, epistatic non-linear interaction discovery, and integration with other omics sets have all been addressed by the ML methods [73]. Thus, these innovative ML algorithms associated with the computational and statistical pipeline of GWAS can be utilised for the genetic performance evaluation of animals under heat-stressed conditions and a better understanding of genetic adaptation. Moreover, the genes identified as candidates for thermo-tolerance can significantly help in strategising breeding programmes by implementing genomic selection and marker-assisted selection (MAS) in the future. However, there have been novel developments in GWAS that rely on ML and AI methodologies, such as DL, but these warrant more investigation in future. 

In recent years, there has been an increase in genome-wide detection of selection signatures/selection sweeps due to advancements in high-throughput technologies. Selection signatures of heat tolerance in Dehong cattle, a Chinese indigenous zebu breed thriving in hot environments, have been investigated, and the results revealed that heat adaptation of an organism may be influenced by genes related to heat shock, oxidative stress response, coat colour, feed intake, and reproduction [76]. Selective sweeps can be found using various techniques, ranging from straightforward applications utilising summary statistics to intricate statistical procedures. However, these statistical models’ propensity for producing erroneous findings when their presumptions are violated is one of their major issues, and this is where the use of models powered by AI becomes evident. Application of ML algorithms that approach the task of identifying selection signatures as a classification problem can be considered. Moreover, researchers can adopt ML to improve these statistical models’ precision and prediction accuracy and detect selection signatures efficiently [77]. Shortly, advanced ML-powered neural networks in the livestock sector can be adopted to efficiently handle vast and complex data to detect accurate selection signals [78]. 

**Figure 3:**
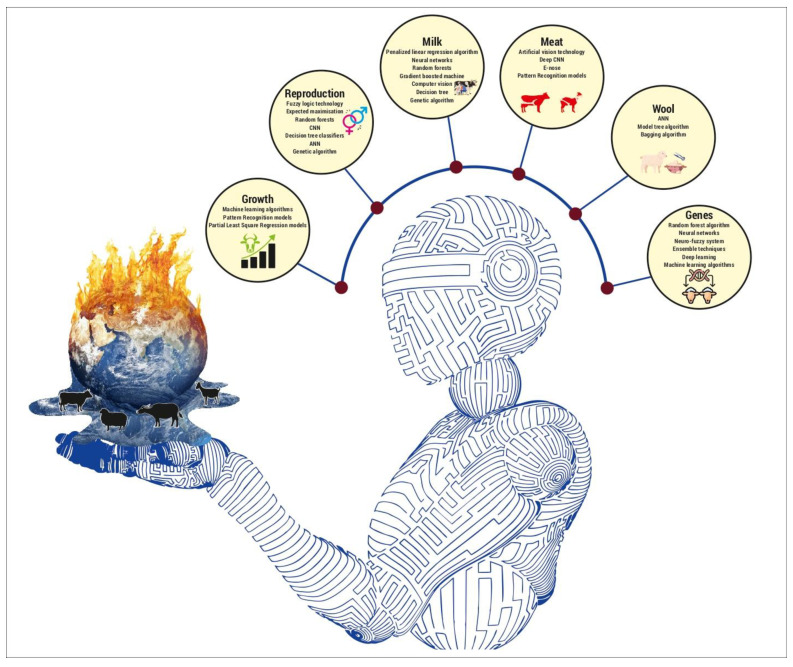
Artificial intelligence technologies adopted in the evaluation of production and genetic performance in ruminant livestock.

Further, significant DL software with the availability of supercomputing using graphics processing unit technology (GPU) has made it possible to integrate environmental variables with multi-omics big data [62]. Thus, there are various applications of AI in genetic evaluation, with a plethora of ML algorithms [62,79] in use and new DL models being validated with further research concerns [80]. Thus, scalable and responsible applications using these technologies can be adopted to achieve optimal performance in heat-stressed conditions and sustain production while selecting features that promote resistance to heat stress. However, AI technologies are yet to be extensively utilised to evaluate the genetic potential of heat-stressed ruminants; nonetheless, the review suggests that the role of AI will become increasingly vital for genetic evaluation. A possible quantum of the literature evidence related to the genetic evaluation of heat-stressed animals has been disclosed. Thus, future studies on AI-based genetic evaluation of heat-stressed animals are warranted, as genetic information can redirect breeding goals. Figure 3 summarises AI technologies adopted to evaluate production parameters and genetic performance in livestock ruminants. 

## 5. AI Technologies for Automated Monitoring of Heat Stress in Livestock

Heat stress in ruminant livestock causes disturbances in their capacity to regulate body temperature and is manifested by behaviour, physiology, and health alterations. These indicators can be utilised as an effective tool to offer timely intervention with heat alleviation measures to suppress heat load. A combination of advanced precision technologies and AI-powered models can be adopted to monitor heat stress in livestock [81] effectively. Table 2 summarises the AI technologies adopted for automated monitoring of heat stress in ruminant livestock based on behaviour, physiology, and animal health data.

### 5.1. Behavioural Monitoring 

Under heat stress conditions, the common behavioural responses in livestock include alterations in the frequency and duration of feeding, drinking, defecation, urination, lying time, standing time, rumination time and shade-seeking behaviour. Other than the above classical heat stress-related behavioural responses, certain behaviours, such as stepping behaviour [82] and postural alterations in major body regions comprising the head [83], can also be considered in the behaviour evaluation process. The behavioural responses vary among various species of ruminant livestock. In this regard, monitoring wallowing behaviour in the case of buffaloes [84] and increased aggressive behaviour, as reported in cattle [85] and goats [86], can be considered. 

One of the most effective non-invasive ways to gauge the effects of heat stress in animals is through monitoring the behavioural reactions, among the primary responses exhibited by animals to combat heat load [87]. Integrating behavioural monitoring devices with AI-enabled technologies can enhance prediction accuracy and act as a time-conserving approach. Further, in several additional domains, big data and ML are being tested as stand-alone approaches or in conjunction with traditional sensors to assess livestock’s adaptive behaviour [88]. Moreover, these advanced approaches can be adopted readily, as they are considered superior since several ML algorithms eliminate subjectivity by considering non-linearity in the data [13]. 

Daily ambient THI values have been established as having a negative correlation with feed intake, and the stressors may not have an immediate influence on this production variable [89]. Moreover, evaluating drinking behaviour wherein heat-stressed animals show increased water intake to maintain evaporative water loss [90] can be helpful. Thus, the detection of feeding patterns is vital in predicting heat stress impacts on growth and other variables, whilst the detection of drinking behaviour can aid in evaluating heat dissipation. In this regard, a low-cost RGB-D (Red, Green, Blue, Depth) camera and a machine vision system based on a deep CNN model is another approach adopted to measure the feed intake of individual animals. The design of this study was such that the data about feed intake was retrieved from RGB and depth images, and the use of DL algorithms was developed using CNN models for identification and intake estimation [91]. To minimise the limitations of conventional behaviour recognition methods, DL techniques have been implemented to monitor the feeding, drinking, active, and inactive behaviours of goats housed in groups using video sequences of a top upper-side view [92]. 

ML algorithms make it possible to determine the number of animals that sustainably graze on a particular pasture at a given moment [88]. Other advances include ML based on the Inertial Measurement Unit (IMU) and optical sensors [93]. Moreover, a study finding suggests the promising use of ML in classifying behavioural responses (lying, standing, and grazing) of goats using a back-mounted 9-axis multi-sensor (tri-axial accelerometer, tri-axial gyroscope and tri-axial magnetometer) with ML algorithms. In the study, from the raw sensor data, over 100 distinct variables were retrieved and two supervised ML algorithms, K-nearest neighbours (KNNs) and decision tree (DTs), were then used to classify the variables [94]. The other methodology described in the study is an integration of the Internet of Things and ML wherein goats were detected using a faster Regional Convolutional Neural Network (Faster R-CNN) to locate animals (resting and walking) and recognise eating and drinking behaviour based on the part of the area beyond food and water lines whilst using the centre of the object bounding box as the representative position of the animals [95].

Using novel machine vision technologies can achieve automated, stress-free, non-contact and economical solutions to meet the needs of animal behaviour monitoring indoors and outdoors using natural characteristics of the animals (shape, colour and movement) to observe their behaviour [96]. In addition, it is suggested that extracting features from video sequences, such as geometric characteristics or colour moment features, is crucial in identifying individual eating or drinking behaviours and establishing precise mathematical models, such as CNN or extended short-term memory networks (LSTM) [4,97]. Moreover, a study finding presenting a comparative analysis of two ML algorithms, including random forest and support vector machine (SVM), in addition to a DL CNN model, based on time series data from a 3-axis accelerometer to classify cattle feeding behaviours (eating, ruminating, and other) can be of practical use, as the results suggest an effectiveness of these techniques in precisely classifying feeding behaviours, paving the way for efficient livestock monitoring [98].

It is well established that excretory behaviour varies depending on different environmental conditions, and, specifically, heat stress significantly affects urination and defecation in livestock ruminants, resulting in a decrease in defecation and urination frequency [99]. Further, the analysis of ruminant faeces can provide valuable clues about the animal’s digestibility and feed intake under heat-stressed conditions. In this regard, a study classified and evaluated dairy cow faeces using RGB image analysis through an AI (CNN) technique that can help evaluate the herd’s nutritional status based on the faecal scores [100]. 

A novel method has been proposed to detect defecation events by fixing a three-axis accelerometer on the tails of three Japanese Black steers in a pasture. To recognise defecation occurrences automatically, the study established six variables (maximum, minimum, and area in convex curve per 30 s for *x*- and *z*-axes). It used quadratic discriminant analysis (QDA) and SVM-ML algorithms, concluding in the use of an accelerometer in effectively detecting defecation events [101]. Further, another study proposed the potential use of non-invasive accelerometer sensors in the effective detection of characteristic back arching during urination and dung events in cows using ensemble ML models to predict urination and defecation events, as well as in the estimation of urination characteristics (frequency and duration) [102]. 

Random forests are ML models that evaluate many regression or classification trees on a trained dataset and determine the best ensemble ML models. Random forest can be employed as it is a robust way to classify activity using accelerometer data, allowing classification accuracy to be tested for specific behaviours [103]. In this regard, a study employed random forest models on the data collected from a rear-mounted tri-axial accelerometer that reliably identifies ewes exhibiting a characteristic squat when they urinate. In the same study, accelerometer data revealed a specific pattern for urination, with a 5 s window providing the best recall and a 10 s window delivering the highest precision. In addition, ‘State’ behaviours (foraging, walking, running, standing, and lying down) were also accurately and reliably detected [104].

Animals during high ambient temperature conditions prefer to stand rather than lie down to maximise the body surfaces accessible to dissipate heat by evapotranspiration, especially through the skin over the underparts of the animal, which is less covered by hair, thus exposing skin to cooling air flow [105]. In this regard, the adoption of advanced, efficient and non-invasive methods of postural behaviour recognition is vital. A study proposes developing a computer-vision-based system that recognises sow behaviours such as lying, sitting, standing, kneeling, feeding, drinking, and shifting and automatically processes and classifies behavioural images effectively [106]. This system can be readily adopted in ruminants, as it is conducive to investigating behavioural changes. 

A DL framework with a detector and faster R-CNN can also be adopted to identify different postures like standing, sitting, sternal recumbency, ventral recumbency, and lateral recumbency [107]. Further, using ML algorithms like KNN, some behaviours were identified by defining walking behaviour as a beat motion for at least 2 s; standing behaviour as sheep standing on their four legs, head up or down; and lying behaviour as sheep lying on the ground with or without jaw movement. In the same study, the results revealed that the combined offline trained classifier and online learning algorithmic classifier approach is useful in the accurate classification of behaviours in sheep presented with changing environmental conditions [108], aiding as a potential system to provide real-time and long-term automated monitoring of behavioural responses. 

The use of the ML model for predicting lying behaviour in dairy cows reared on pastures and indoors has been investigated. The study used the data collected from a collar-based prototype and cameras in parallel to develop an ML model with efficient prediction of lying behaviour [109]. A research result disclosed the methodological framework for successfully predicting six behaviours (grazing, walking, ruminating while standing, ruminating while lying, resting while standing, and resting while lying) in dairy cows using three-dimensional accelerometer data. In the study, four ML methods (eXtreme Gradient Boosting, random forest, SVM, and Adaboost) were compared with the consequent application of the Viterbi algorithm, and the findings reveal a superior prediction capacity using the XGB algorithm followed by Viterbi smoothing, with its potential use for decision-making and monitoring purposes [110]. Another study applied an ML technique named Viola–Jones algorithm in modelling classifiers and validating cow standing and feeding behaviour detectors [111]. Thus, various studies have warranted the accuracy and efficiency of AI-enabled tools in monitoring postural behaviour responses.

Feeding and rumination patterns have been documented to change in animals under heat-loaded conditions in order to reduce metabolic heat production, thus serving as a marker of heat stress [112]. However, manual observations are laborious, time-consuming, subjective, and prone to individual variance, making adopting AI-powered tools readily acceptable. A study involved using a novel monitoring system using CNN-based DL models that proved efficient with 95%, 98%, and 98% average accuracy, recall, and precision, respectively [113]. Another research effort revealed the use of data collected from an accelerometer/gyroscope sensor attached to the ear and collar of sheep in developing a classification system for grazing and rumination behaviour using four ML algorithms (random forest, SVM, KNN and adaptive boosting). This study’s finding suggests its use in the development of an automatic device for monitoring grazing and rumination behaviour [114]. Similarly, another research work was extended to dairy cows raised in barns, wherein a triaxial acceleration sensor collected data and classification of feeding, rumination and other behaviours was carried out using three ML algorithms, including KNN, SVM, and a probabilistic neural network, concluding its practical application due to high accuracy [115].

Another study reveals the significant use of collar-mounted accelerometer data in classifying feeding and ruminating behaviours using decision-tree algorithms that have been found to perform similarly to SVM and Rumiwatch noseband sensors in terms of accuracy, thus suggesting their implementation on the sensors and the online measurement of ingestion behaviours [116]. Further, a study proposed a new algorithm called the Chew–Bite Intelligent Algorithm (CBIA) based on concepts from pattern recognition and ML areas for classifying masticatory events from the acoustic signals. This work employed conventional and advanced ML approaches to classify three types of masticatory events: chew, bite, and chew–bite, demonstrating their robustness if practically adopted [117]. Thus, many studies adopting varied ML algorithms [118,119] have been proposed to assess rumination behaviour in ruminant livestock. 

Thus, the use of AI-powered tools in behavioural monitoring can be exploited to understand the adaptive mechanism that can aid in strategising solutions. Further, such efforts to establish behavioural adaptation in animals may reveal key biological indicators that allow us to identify the most adapted animal, and such markers can be used in breeding programmes to develop a thermo-tolerant breed through marker-assisted selection [89]; moreover, based on the evidence, it can be suggested that AI-assisted tools can be promising in the evaluation of behaviour and thermal status of heat-stressed livestock under field conditions. 

A study on dairy cows aimed at finding non-invasive measures of assessing heat stress using AI tools. This study demonstrated the application of AI tools as an integrated approach to measure, predict, and evaluate heat stress [120]. The application of assessing heat stress responses like altered behaviour (drinking, eating, lying, standing-in, and standing-out) using a DL-based model; body surface temperature using ghost and channel attention modules of neural networks; and respiration rate using computer vision along with the use of four ML algorithms to predict respiration rate, vaginal temperature and eye temperature, has been demonstrated in the study [120]. Another study in dairy cows reflects the use of ML-approaches for automatic characterisation of behavioural phenotypes pertinent to thermo-tolerance [121]. The study prioritised brush to use and drinking behaviour, considering them to be integral responses to maintaining homeostasis during heat stress conditions. This study established the application of AI tools in quantifying behavioural responses to heat stress, which are otherwise laborious to quantify using traditional methods. Moreover, this approach opens up a provision for producers to make informed decisions [121]. Further, a study assessed the adoption of AI tools in determining the onset of heat stress in dairy cows [122]. This study involved using DL-models for recognising animal behaviours and computing the behavioural indicators of heat stress in a herd basis [122]. These findings concluded using AI technologies as a non-invasive, low-cost heat stress alert tools for dairy cows. 

In a study on lactating heifers, automated monitoring devices such as smart tag leg and smart tag neck were used to document the behavioural activity of heifers and established that the late-gestation exposure to heat stress affects the daily time budget of first-lactation heifers during both the pre-and postpartum periods. These sensor-based devices were very effective in assessing the influence of heat stress on heifers’ behaviour [123]. In another study on dairy cows to determine the season on behaviour, leg tags were used for recording daily activities, lying time, and the number of steps and standing bouts, and neck tags were used to measure eating and rumination time [124]. This study provided a better understanding of how different seasons affect the daily time budget of lactating dairy cows and may contribute to developing more effective management strategies to decrease the adverse effects of heat exposure [124]. 

### 5.2. Physiological Monitoring 

Physiological alterations are among the animal-based indicators of excessive heat load, and they help the animals cope with the stressful environment. Respiration rate (RR), pulse rate (PR), rectal temperature (RT), sweating rate (SR), panting rate, and skin temperature (ST) are the cardinal physiological variables that are altered in heat-stressed animals [125,126]. Thus, quantifying physiological responses using advanced techniques is crucial for effective heat stress assessment and management. 

Research findings report the use of ML algorithms (penalised linear regression, random forests, gradient-boosted machines, and neural networks) to assess the impact of several environmental heat stressors, including air temperature (AT), relative humidity (RH), solar radiation (SR), wind speed (U), and solar radiation on the physiological responses (RR, ST and vaginal temperature (VT)) of dairy cows. The same study suggested that an approach using ML algorithms removes subjectivity and helps in ranking the heat stressors that would help farmers implement evidence-based remedies before the expected stressful environmental circumstances occur [13]. An implantable thermometer has been proposed to monitor core body temperature for a longer duration, wherein the study used ML techniques to predict core body temperature from the subcutaneous temperature [127]. 

Another captivating study established a combination of infrared thermography (IRT) and ML techniques in predicting heat stress non-invasively in sheep. The ANN model developed in the study revealed the best performance and accuracy when utilising IRT to predict rectal temperature and detect heat stress with minimal restraining stress [36]. Further, research findings revealed the implementation of computer vision methods in estimating heart rate, RR and abrupt movements, and using ML models to predict eye temperature, milk production, and quality. The study concluded that this technique can be deployed in conventional dairy farms and can aid in automatically identifying and manipulating stressful environments within the farm [44]. Similarly, other research results propose the efficient use of a neural model, accepting IRT, dry bulb temperature, and wet bulb temperature as inputs to estimate RT. In addition, the study compared a regression model with the neural model and suggested strongly with evidence that the neural model has an excellent predictive ability in comparison to the regression model [128]. Similarly, a study finding indicates that ANN-based models developed using defined weather and physiological variables predict RR and RT better than the linear regression models and enable individual assessment of the thermal status of an animal [129]. Another study in dairy cows predicted RT and RR as a function of dry-bulb air temperature and relative humidity. Moreover, upon a comparison of different models in the study, it was found that neuro-fuzzy networks and regression fared equally, while the ANN model demonstrated the best performance [130]. Another study compared five different models to predict RR based on dry bulb temperature, dew point temperature, solar radiation, wind speed, and breed. The study concluded that both the neural network and the fuzzy inference system performed well in predicting RR [131]. However, recently, a study in dairy cows has been carried out to overcome the limitation of using limited variables to predict physiological responses, as the resulting models had poor predictive capability. In this regard, the study developed ML models utilising comprehensive variables to predict physiological responses better. The study employed random forests, gradient boosting machines, ANNs, and regularised linear regression to predict RR, VT, and eye temperature with 13 predictor variables from three different dimensions, such as production, cow-related, and environmental factors [132]. In another study, PVDF flexible piezoelectric sensors were used to record respiratory rhythm in Hu sheep, and the technology provides potential technical support for future health monitoring and early prediction of diseases in large farm animals [133]. 

Thus, several studies adopted ML techniques to predict physiological indicators of heat stress in livestock ruminants. This evidence suggests the practical application of advanced AI-enabled technologies to predict physiological alterations or potential circumstances of when heat stress will occur in livestock animals. This would enable more effective implementation of management practises while offering a non-invasive way to shield animals from extreme environments.

A study in dairy buffaloes demonstrated the evaluation of thermoregulatory responses (skin surface temperature and respiratory rate) and indices related to environmental variables using an ML approach, wherein an ANN with a single layer was used for the analysis [134]. The study established that the thermal comfort of the animals can be predicted using the skin surface temperature. Thus, this study stresses the possibility of assessing animal thermal response [134]. Another study in feedlot heifers evaluated predictive models (regression models, fuzzy inference systems and a neural network) that can be used to forecast stressful environments, aiding in the adoption of preventative intervention by livestock producers [131]. The study describes that the model using a fuzzy inference system predicted the respiration rate in accordance with environmental data. 

### 5.3. Animal Health Monitoring 

It is well established that heat stress is a multi-faceted and ongoing challenge impacting livestock production. In this regard, animal health monitoring is a crucial indication of the animal’s welfare state. Moreover, heat stress increases an animal’s vulnerability to various diseases by suppressing the immune and endocrine systems [135]. The increase in temperature has also been reported to bring about physiological alterations that negatively impact the ruminants, resulting in an increased risk of metabolic disorders and health problems [112]. In addition, the risk of emerging and re-emerging pathogens and disease vectors is another facet of climate change-associated temperature extremes [136]. Livestock diseases bring about a diverse economic impact directly by causing production losses and indirectly as treatment costs [88]. In addition, extreme heat stress is accompanied by increased morbidity and mortality rates in livestock. Evaluating disease dynamics and animal adaptability will be critical to their resilience [137], and thus it is vital to adopt robust animal health monitoring tools. 

Recently, increasing interest in data mining for purposes like enhanced disease diagnosis has been driven by expanding animal and environmental data gathered automatically by sensors for real-time monitoring. ML methods that go beyond traditional regression are believed to be effective at this task. A study details the application of IRT and ML technologies, along with the principles of capturing thermal images and parameter data extraction, in addition to revealing the development and research progress of IRT technology in animal health evaluation, with a primary focus on bovine disease detection, such as mastitis, lameness, respiratory diseases, and so on, but also on indicators for assessing health status, such as physiological characteristics, stress, temperament, and oestrus. The study concentrates mainly on the tasks and applications of ML and DL algorithms in thermal infrared imaging data processing [138]. In this regard, the practical application of ML and DL algorithms in detecting heat stress-related diseases must be scrutinised. Another research finding discloses that the behavioural responses recorded with wearable sensors can be classified using an AdaBoost ensemble learning algorithm. This study was exclusive, as it employs ML approaches for multi-class behaviour identification and behaviour quantification in calves, thereby having plausible potential in animal health and welfare assessment [139].

Many studies have uncovered that under hot environmental conditions, the incidence rate and pathogen count of mastitis increase due to favourable growth conditions at higher temperatures while reducing the phagocytosis and immune response of the animals [140,141]. Hence, evaluating the aetiology, pathophysiology, and economic effects of mastitis using sophisticated methodologies is critical in dairy farming. Considering this, a study exposes the use of field surveys and the Dairy Herd Improvement Association (DHIA) dataset to classify the causes of bacterial mastitis in dairy cows using the ANN model [142]. Similarly, many studies have validated the effective use of neural networks in the automated detection and prediction of mastitis [143,144,145]. For instance, another study discloses the effective use of a recurrent neural network (RNN) model for detecting clinical mastitis in dairy farms with automated milking systems by integrating various variables such as milk traits, behavioural characteristics, cow traits, and environmental variables [146]. Moreover, in this golden era of precision dairying, a rapid and automated technique for disease identification brought about the use of IRT as an emerging tool for disease prediction. In addition, algorithms like KNN, SVM, random forest, and CNN have been adopted for real-time mastitis detection using thermographic images [147]. Similarly, the detection of dairy cow mastitis by DL technology combined with a comprehensive detection method, thermal infrared thermography, has been proposed to be highly accurate [148]. 

Studies have established the use of SVM for early mastitis detection [149,150]. Furthermore, a study finding uncovers the efficient use of two mastitis detection models, ANN and adaptive neuro-fuzzy interface systems (ANFIS), for the prediction of subclinical mastitis with inputs on lactation rank, milk yield, electrical conductivity, average milking duration, and season [151]. Moreover, the adoption of the ANN model, which uses electronic 3D motion detectors to detect the early symptoms of mastitis, has also been disclosed as being effective [152]. 

It is well known that pests carried by vectors, including flies, ticks, and mosquitoes, are affected in number and distribution by variations in precipitation and global warming due to climate change. Furthermore, in hot weather conditions, there is an increase in the likelihood of disease transmission between hosts [153]. Thus, comprehending the disease dynamics is vital in evaluating animal heat stress-related health problems. AI has been reported to be effective in predicting pathogens related to food-borne diseases using many popular methods, including decision trees, random forests, KNN, stochastic gradient descent, and extremely randomised trees, as well as an ensemble model incorporated in all these systems [154]. Moreover, ML algorithms have been trained using outbreak datasets and AI models in forecasting disease outbreaks based on transmission dynamics [155]. Using AI technologies poses multiple benefits in human medicine, including monitoring and prediction [156]. These cutting-edge technologies can be extended to the veterinary field to efficiently evaluate disease patterns. 

A study developed a cost-effective parasite diagnostic system wherein the faecal samples prepared can be imaged and analysed using a trained CNN to robustly identify egg species and egg counts with good accuracy and excellent performance [157]. Other research results reveal that DL-powered Caprine parasite detection aids in enhancing animal health [158]. In addition, automated learning techniques have been well utilised in vector-borne disease modelling, aiding in early decision support [159]. AI-enabled technologies thus significantly impact the early detection and prevention of diseases. They forecast disease outbreaks by evaluating big datasets from varied sources, allowing for proactive disease control measures to ensure better survival rates [160]. Thus, varied heat stress-induced pathology can be diagnosed and predicted by adopting AI tools.

**Table 2:** AI technologies for automated monitoring of behaviour, physiology, and animal health, which could be used either directly or indirectly to assess heat stress in ruminant livestock.

Objective	Monitored Response	Key AI-Technology	Advantages	Disadvantages	Reference
Behavioural monitoring	Feeding	Machine vision system based on deep CNN	Accuracy, Cost-efficient, feasibility and adaptability of the model	Requires training of model with highly diverse data	[91]
	Feeding and drinking	Deep learning	Detection speed, Accuracy, cost-efficient	Invalid frames increases error rates	[92]
	Feeding	Machine learning	Excellent prediction accuracy	Difficulties in behaviour discrimination	[88]
	Feeding	Machine learning	Accuracy, specificity and precision	Low sensitivity	[93]
	Grazing, lying and standing	Machine learning algorithms: KNN and decision tree (DT)	DT algorithm tends to have low computational cost, high computation capacity and low-energy consumption	Low predictive accuracies, Requires large dataset	[94]
	Feeding, drinking, resting and walking	Integration of Internet of Things and machine learning (Faster R-CNN)	Higher detection accuracy and reliability	Low detection speed	[95]
	Feeding, drinking, lying, locomotion, aggression, reproductive behaviour	Machine vision system	Non-contact, non-stress, cost-effective, commercially applicable		[96]
	Feeding and drinking	CNN and long short term memory networks	Recognition accuracy	Influence of animal factors and pen conditions influences performance, False classification	[4,97]
	Feeding	Random forest, support vector machine and deep CNN	Precision, accuracy	-	[98]
	Defecation	CNN	Adaptability, high training and validation accuracy	Requires large dataset for strengthening the model	[100]
	Defecation	Quadratic discriminant analysis and support vector machine	High accuracy	Low discrimination power	[101]
	Urination and defecation	Machine learning	Low-cost, non-invasive, faesibile, high precision, accuracy and sensitivity	-	[102]
	Urination, foraging, walking, running, standing and lying	Random forest model	Sensitive, high recall and precision	-	[104]
	Standing, lying and walking	KNN	Faesibility, high accuracy and specificity	Low recall and precision	[108]
	Lying	Machine learning	High sensitivity, specificity and accuracy	Low prediction	[109]
	Grazing, walking, ruminating while standing, ruminating while lying, resting while standing and resting while lying	Machine learning algorithms: eXtreme gradient boosting, random forest, support vector machine and Adaboost	Excellent prediction accuracy	Low discrimination capacity	[110]
	Standing and feeding	Viola–Jones algorithm	High accuracy and sensitivity	Expensive, low feasibility	[111]
	Rumination	CNN-based deep learning	High accuracy, recall and precision	-	[113]
	Grazing and rumination	Machine learning algorithms: random forest, KNN, support vector machine and Adaptive boosting	High accuracy and performance	Misclassification errors, requires large dataset	[114]
	Feeding, rumination and other behaviours	Machine learning algorithms: KNN, support vector machine and probabilistic neural network	High specificity, precision, recall and accuracy	Misclassification errors	[115]
	Feeding and rumination	Decision tree algorithm	High specificity, precision, and sensitivity	-	[116]
	Masticatory events	Pattern recognition and machine learning	High recognition rate, precision and recall	High computational cost	[117]
	Rumination	Machine learning	High precision, recall, specificity, and accuracy	Low sensitivity	[118,119]
Physiological monitoring	Respiratory rate, skin temperature, vaginal temperature	ML algorithms: penalized linear regression, random forest, gradient-boosted machine and neural networks	High accuracy and prediction	-	[13]
	Core body temperature	Machine learning	Good prediction and accuracy	-	[127]
	Rectal temperature	ANN	Cost-effective, non-invasive, high accuracy and performance		[36]
	Heart rate respiratory rate and eye temperature	Machine learning, computer vision	High prediction accuracy, feasibility and cost-effective	-	[44]
	Rectal temperature	Neural networks	Good predictive ability and performance	-	[128]
	Respiratory rate and rectal temperature	ANN model	Individual level assessment, good predictive ability and accuracy	Requires large database to improve efficiency	[129]
	Respiratory rate and rectal temperature	ANN and neuro-fuzzy network	Good performance and predictive capacity	-	[130]
	Respiratory rate	Fuzzy interference system and neural networks	Fuzzy interference system has good performance	Low prediction accuracy	[131]
	Respiratory rate, vaginal temperature and eye temperature	Random forest, gradient boosting machines, ANN and regularised linear regression	High prediction capacity and reliability	-	[132]
Animal health monitoring	Bovine disease detection (mastitis, lameness, respiratory diseases and so on) and indicators of health status (stress, temperament and oestrus)	Machine learning and deep learning algorithms	Non-invasive, good performance	-	[138]
	Behaviour based animal health and welfare assessment	Adaboost ensemble learning algorithm	High accuracy, low overestimation	Labour requirements to label behaviours, implement multi-class quantification methods for behaviours with low prevalence rate, sampling frequency required	[139]
	Bacterial mastitis	ANN model	Good performance	Low prediction capacity	[142]
	Mastitis	Neural networks	High classification rate, sensitivity, specificity and accuracy	Requires field validation, low predictive capacity	[143,144,145]
	Clinical mastitis	RNN model	Effective performance, good sensitivity and specificity		[146]
	Mastitis	KNN, support vector machine, random forest and CNN	Non-invasive, rapid and cost-efficient	Low accuracy due to limited data	[147]
	Mastitis	Deep learning	High detection accuracy, sensitivity and specificity	Extra accessories required to improve accuracy	[148]
	Mastitis	Support vector machine	High sensitivity	Average specificity, low performance	[149,150]
	Subclinical mastitis	ANN model, adaptive neuro-fuzzy interface system (ANFIS)	ANN model has high sensitivity and specificity	High prediction error rate	[151]
	Mastitis	ANN model	High accuracy, sensitivity and specificity	Low classification capacity	[152]
	Food-borne diseases	Decision tree, KNN, stochastic gradient descent, extremely randomised trees and ensemble model	Good predictability, high accuracy, recall and precision of ensemble model	-	[154]
	Forecast disease outbreaks	Machine learning algorithms	Good performance	Data integration, need for more resources	[155]
	Parasite diagnostic system	CNN	Cost-effective, robust and simple	-	[157]
	Parasite detection aid	Deep learning	Precise and rapid classification	-	[158]

## 6. Application of AI-Based Technologies for Heat Stress Management in Ruminant Livestock

Regular animal husbandry management practises have been scrutinised and refined in recent years due to drastic changes in weather patterns. From this perspective, the advent of AI-powered tools has plausibly penetrated animal farming in modulating management measures under varied and adverse environmental conditions. These potent and solidly built robust technologies have revolutionised the animal farming sector comprehensively in all aspects related to management. AI is widely used in livestock farm management to gather and evaluate data to improve decision-making and streamline farming processes. The AI systems may track and assess animal behaviour, health indicators, and level of productivity using sensors, IoT devices, and data analytics [9]. Collecting real-time monitoring data enables a multi-model behaviour-based heat stress alarm model for strategising effective management practises and mitigation interventions [90].

The most effective short-term action plans for an impending heat wave event may involve proactive interventions based on climate forecasts and adjustments depending on actual environmental demand. Real-time monitoring, identification, and isolation of individual animals for heat vulnerability are now feasible because of advancements in animal monitoring technologies. Moreover, by turning on a single- or multi-sensor whole-farming network using IoT, it is also achievable to automate the mitigation procedure only for the isolated animal [161]. In addition, a recent innovation of an integrated technique, the Artificial Intelligence of Things (AIoT), that incorporates AI and IoT, is adopted readily as it can sense changes in the barn environment and animal diseases. It can swiftly and automatically respond to problems without human assistance, thereby improving efficiency and productivity [162].

A study has proposed a heat stress monitoring and mitigation protocol based on on-animal and off-animal technologies that monitor the heat stress response, which transfers information to the central data repository that refers the information singly or in combination with parallel data like environmental parameters to the processing system. The study disclosed that the processing unit works in the following two ways: responds for the group of animals by automatically activating heat abatement measures (water spray, air cooling, and other environmental modifiers) or responds for individual animals by creation of virtual fencing (VF) around cooling zones only for the animals susceptible to heat stressor or isolation based on GPS-VF to a separate cooling zone. Moreover, the data repository of this robust system can be utilised in future for the evaluation of individual animal responses during heat wave episodes, thereby aiding in the identification of thermo-tolerant animals that could assist in breeding programmes [90]. Thus, incorporating these automated and robust systems could help achieve effective heat stress management conditions favourable for improving efficiency with optimal production. 

A typical occurrence in many habitats is the presence of multiple stressors, which will probably intensify due to climate change [2]. One of the stressors associated with a hot environment is nutritional stress, as the availability of pastures is significantly limited, especially in the summer when droughts are widespread. Pasture availability is affected in terms of quantity and quality, predisposing animals to severe nutritional deficits as a result. Hence, advanced tools are applied in animal management practises to monitor pastures effectively. Extensive research on pasture monitoring has created sophisticated statistical and ML/DL models to forecast characteristics related to fodder quality and quantity. Moreover, it is found that the fundamental regressive technique for assessing pasture conditions is supervised learning [163]. The AI-based recognition system can rate the pastures, which is enabled by the trained algorithm to generate valid recommendations [164]. However, adopting supervised learning is hampered mainly by the need to gather a significant number of samples for laboratory analysis to train and validate the data for models [163]. Moreover, studies have established that, by employing computer vision and based on the registered image, farmers can estimate the amount of pasture available for the ruminants; additionally, based on the body weight of the animal, they can assess if these estimates match the recommended daily allowance [165].

**Figure 4:**
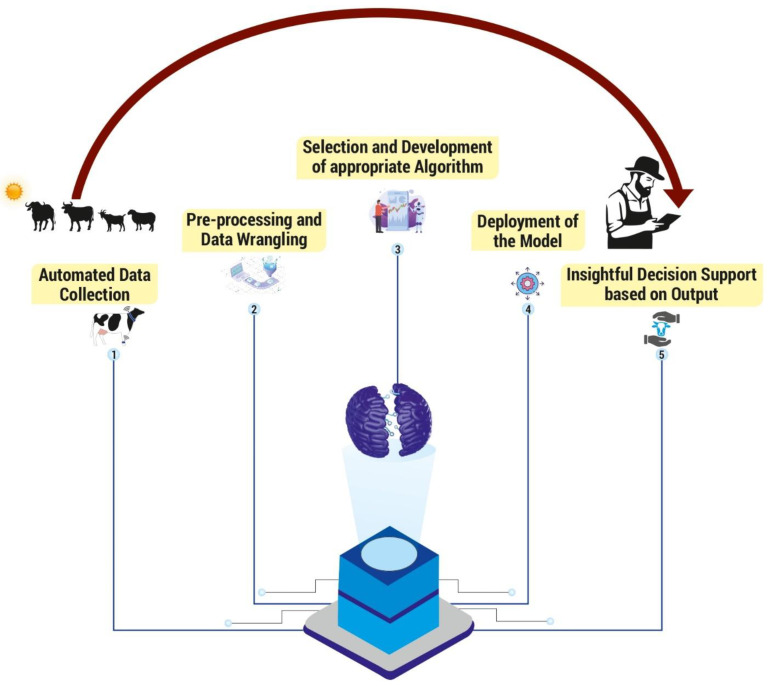
Transmission pipeline of AI-enabled tools for heat stress management in ruminant livestock production systems. The process involves automated data collection (behavioural, physiological, health and environmental data), pre-processing and data wrangling (integration of input data and data preparation), selection and development of appropriate algorithm (build, train and evaluate model), deployment of the model (integrating model in production environment, prediction/classification) and insightful decision support based on output (real-time welfare assessment, early stage warning and forecasting).

Transportation, an important animal husbandry practise also predisposes animals to stress during summer, affecting the health and welfare status of the animals. To address this issue, a study disclosing predictive models integrated with environmental variables and stress markers can be developed using supervised learning networks to forecast transport stress in animals [166]. Moreover, other effects due to hyperthermia include serious impairment of reproductive processes in females by causing adverse effects on the oestrus cycle. In this respect, effective oestrus identification can aid in effectively managing the herd. Many AI-based models have been developed to detect oestrus early in ruminant livestock [167,168,169,170]. A recent advancement has uncovered a numerical modelling approach to project the future heat stress risk in animal husbandry systems. The study proposed an ANN model powered by an ensemble of regional climate model projections with three different greenhouse gas concentration scenarios that evaluated heat stress events and estimated economic and environmental impacts [171]. These cutting-edge research findings reveal the extent of technological progress in the livestock sector. Another invading research reports an ML-based heat stress scoring system that aids in evaluating heat stress severity in cows by utilising different heat abatement techniques [81]. Thus, various innovative developments in AI pave the way for convincing and adaptable management opportunities for the successful mitigation of heat stress in animals. Figure 4 illustrates the transmission pipeline of AI-enabled tools for heat stress management in ruminant livestock production systems. Lastly, with the help of the Internet of Things, big data, artificial intelligence, etc., non-contact sensing technologies can better play their role in assessing animal welfare accurately, and, thus, these agri-sensing technologies could play a significant role in the future by promoting precision livestock farming [172]. 

## 7. Using of Artificial Intelligence for Promoting Climate-Smart Farming in Livestock Extension 

Livestock extension in developing countries primarily focuses on sharing valuable information with livestock farmers, capacity-building activities, and technical advice with a focus on improving their livestock-based livelihoods and enhancing the productivity of their farm animals for better incomes and well-being in the households. Specific livestock extension services are mandated to facilitate encounters with farmers on farming technologies and solutions, such as breeds, feeds, vaccines, disease prevention and control practises, farm equipment, and packages of practises, and encourage them to put them into practise, etc. Furthermore, extension services also provide dynamic information services, such as weather-based advisory services, market information, farmers’ demand-based information services, and forward and backward linkages. The livestock extension services in developing countries of the African continent have facilitated uptake technologies among farmers and enhanced the production and returns from livestock farming [173,174,175,176,177]. Similar to these trends, the impact of livestock extension work has been reported in the South Asia region [178,179]. Furthermore, livestock extension acts as an interphase between research labs and farmers, acting as a feedback system on technologies and also facilitating the identification of researchable issues. 

The livestock extension services in third-world countries are primarily provided through public sector organisations such as the Ministry of Animal Husbandry/Agriculture through their organisational networks at the grassroots level. Public sector organisation activities are supplemented with non-governmental organisations, farmer’s collectives, commodity-based producer organisations, and the private sector. Public sector investment in livestock extension systems is in a declining phase, resulting in a shortage of human resources and budgets across third-world countries. The reach of the private sector, NGOs, and farmers’ collectives are restricted to regions with in-country and commodities. This limits farming communities’ technology uptake. In the case of India, the extent of adoption of livestock farming technologies ranges from 47 to 55% [180,181], and a productivity gap of 30 to 72% exists [182]. In addition to these challenges, in the context of climate change, livestock production needs to be made sustainable, with fewer GHG emissions. Also, livestock farming needs to be adapted to the changing climate. This scenario demands building climate-smart livestock extension services. 

Climate-smart livestock extension services may leverage information communication technologies (ICT), including Artificial Intelligence (AI) and machine learning (ML), to address the challenges within and outside the livestock extension system. Deployment of ICT tools in livestock extension is in progress. Developmental agencies have been investing in development applications for feed rationing, breeding, monitoring of animal production parameters, education of farmers, identification of animals, etc., focusing on individual farmers using smartphones. However, these ICT applications face challenges in terms of demand for multiple language services, localisation of information content, and ICT infrastructure challenges, including issues associated with internet connectivity and access to smartphones. Thus, the enrobing of communication tools with conventional service delivery systems will likely increase access to information in the farming community. 

Securing information on local farming situations, farmers’ practises, and resources is mandatory in developing localised content. Secondly, after securing the above information, there is a demand to generate advisory information as the forecast for farmers. Thirdly, the generated information must be shared with farmers with limited ICT infrastructures and tool access. To address these challenges, the Internet of Things, AI (Artificial intelligence) and ML (machine learning) pave the way. The recent development of non-invasive methods to quantify animals, physiologic conditions, and climatic stress on animals through IoT devices helps to relate the external environment with animal physiological parameters [87,183]. Periodically captured animal and environmental data can be subjected to data ingestion, data pre-processing, and the building of an AI model. The ML algorithm can be trained to estimate indices such as thermal stress for livestock (ITSC) and an index for the time spent in the shade (ITS), and, for these kinds of indices, weather-specific advisory elements can be generated. Using this model, customised livestock advisory information, forecasts, and alerts can be created. These AI models can be evaluated and deployed along with conventional extension systems as complementary models to enhance the information sharing process. Specifically, near real-time advisory services can be generated for clusters of villages by engaging with local/government farms for data generation. The IoT-enabled advisory system can be integrated with livestock extension services for real-time data-based advisory services for the farming community. The integration paves the way for reaching out to farmers who have limited access to ICT infrastructures, smart mobile phones, computer literacy, etc. 

## 8. Conclusions

The conventional system of heat stress management using data based on visual observations or climatic indices is impractical, laborious, and prone to human error. In contrast, the speeded-up technological development and digital metamorphosis have led to drastic progress, introducing real-time, autonomous, and efficient systems. In this respect, the rapid progress of AI with its new applications has invaded the livestock sector through the essential development of intelligent systems that can replicate human abilities like learning, problem-solving, and decision-making. This revolutionary technology has significantly penetrated animal farming systems to enable AI where data management is impossible manually. The literature provides evidence of consistent innovations of AI models in animal science with a positive influence and is suggested to be pervasive in terms of its potential to transform the way livestock producers operate. The diverse disciplines of AI comprising specialised systems and algorithms have been implemented in the livestock sector for varied applications, including heat stress management. The precision, accuracy, and performance of the proposed models have also been convincing for scalable applications in effective heat stress mitigation in ruminant livestock. The researchers and livestock producers reconsider the integration of data, analysis, and execution of conclusions by adopting AI-powered techniques in farm management to warrant decision support in this alarming climate change scenario. AI has driven several advances in the performance evaluation, the monitoring and management of ruminant livestock, massive developments in data collection, storage, and analysis, and extreme sophistication in analysis and decision-making. These emerging AI technologies have the potential to tackle challenges effectively and offer improved monitoring and evaluation of stressors associated with high environmental temperatures. Moreover, in the face of drastic climate fluctuations, robust and automated monitoring and assessment tools lay the foundation for sustainability in ruminant livestock production. 

## 9. Future Perspectives

Considering the emerging adoption of AI-enabled techniques in the livestock sector at the current time, the reported challenges and limitations associated with AI are overlooked. Primarily, the size of the recorded datasets required to train the models is small, and thus, the implementation of AI tools in small livestock farms has been challenging and demands a solution. Big data storage, transmission, compression and feature extraction require improvement for the efficient use of these techniques in animal farming systems. Further, a cross-functional and interdisciplinary team involving data analysts, animal science experts, and subject specialists is vital for developing AI models according to the farming conditions to improve efficiency and productivity in varied aspects. Advanced methods for analysing vast amounts of heterogeneous data need to be developed in close cooperation with experts in computer science, engineering, mathematics, statistics, and the livestock industry. Further, the security of the data collected is also a concern, as adulteration and hacking issues need to be restrained. Adopting new and developing digital technologies in farming systems is crucial for an integrated development with AI-tools. This would result in productive and successful applications that are simple to scale up to production. Despite these challenges, the field of animal science is well positioned to benefit from advances in AI and efficiently address the challenges associated with climate change. Big data applications for analysing alterations in animal behaviour associated with hot environments and assessing welfare status will become the mainstream research priorities in the near future. Therefore, addressing all the above-listed constraints is the need of the hour in regard to revolutionising livestock farming involving AI applications to realise the dream of achieving sustainable livestock production to feed the growing human population by 2050. 

## Figures and Tables

**Table 1:** Economic values of different ruminant livestock.

Ruminant Species	Economic Traits	Economic Value
Cattle	Growth Milk production Meat production Reproduction Immune status Draught Power Manure	Sale of milk Sale of milk products Sale of meat Sale of meat products Sale of calves Improved health and more production Ploughing the agriculture field Sale of manure/fertiliser
Buffaloe	Growth Milk production Meat production Reproduction Immune status Draught Power Manure	Sale of milk Sale of milk products Sale of meat Sale of meat products Sale of calves Improved health and more production Ploughing the agriculture field Sale of manure/fertiliser
Sheep	Growth Milk production Meat production Reproduction Immune status Draught Power Manure	Sale of meat Sale of meat products Sale of lambs Sale of milk Sale of milk products Improved health and more production Sale of manure/fertiliser
Goat	Growth Milk production Meat production Reproduction Immune status Draught Power Manure	Sale of meat Sale of meat products Sale of kids Sale of milk Sale of milk products Improved health and more production Sale of manure/fertiliser
